# Azithromycin and the microbiota of cystic fibrosis sputum

**DOI:** 10.1186/s12866-021-02159-5

**Published:** 2021-03-30

**Authors:** Nicole Acosta, Christina S. Thornton, Michael G. Surette, Ranjani Somayaji, Laura Rossi, Harvey R. Rabin, Michael D. Parkins

**Affiliations:** 1 Department of Microbiology, Immunology and Infectious Diseases, University of Calgary, 3330 Hospital Drive, NW, Calgary, Alberta Canada; 2 Department of Biochemistry and Biomedical Sciences, McMaster University, Hamilton, Ontario Canada; 3 Department of Medicine, University of Calgary, 3330 Hospital Drive, NW, Calgary, Alberta Canada

**Keywords:** Cystic fibrosis, Macrolides, Azithromycin, Microbiome, *Stenotrophomonas*

## Abstract

**Background:**

Azithromycin is commonly prescribed drug for individuals with cystic fibrosis (CF), with demonstrated benefits in reducing lung function decline, exacerbation occurrence and improving nutrition. As azithromycin has antimicrobial activity against components of the uncultured microbiome and increasingly the CF microbiome is implicated in disease pathogenesis – we postulated azithromycin may act through its manipulation. Herein we sought to determine if the CF microbiome changed following azithromycin use and if clinical benefit observed during azithromycin use associated with baseline community structure.

**Results:**

Drawing from a prospectively collected biobank we identified patients with sputum samples prior to, during and after initiating azithromycin and determined the composition of the CF microbial community by sequencing the V3-V4 region of the 16S rRNA gene. We categorized patients as responders if their rate of lung function decline improved after azithromycin initiation. Thirty-eight adults comprised our cohort, nine who had not utilized azithromycin in at least 3 years, and 29 who were completely naïve. We did not observe a major impact in the microbial community structure of CF sputum in the 2 years following azithromycin usage in either alpha or beta-diversity metrics. Seventeen patients (45%) were classified as Responders – demonstrating reduced lung function decline after azithromycin. Responders who were naïve to azithromycin had a modest clustering effect distinguishing them from those who were non-Responders, and had communities enriched with several organisms including *Stenotrophomonas*, but not *Pseudomonas*.

**Conclusions:**

Azithromycin treatment did not associate with subsequent large changes in the CF microbiome structure. However, we found that baseline community structure associated with subsequent azithromycin response in CF adults.

**Supplementary Information:**

The online version contains supplementary material available at 10.1186/s12866-021-02159-5.

## Background

Within cystic fibrosis (CF), lung disease is responsible for the majority of morbidity and mortality associated with the genetic disease [1]. A worsened disease status has been associated with airways infection with several organisms such as *Pseudomonas aeruginosa*, *Burkholderia cepacia* complex (BBC), *Stenotrophomonas maltophilia*, *Achromobacter* spp., and *Mycobacteriodes abscessus* [2, 3] - cultivated through traditional techniques. However, a large body of evidence using culture-independent approaches has now established that CF airways represent polymicrobial infections, with the residing community of organisms termed as the CF microbiome. Data from our group has previously demonstrated that the CF sputum microbiome is a better predictor of disease course than traditional cultured pathogens [4], and baseline community structure associates with response to CF disease modifying therapies [5–7].

One of the most frequently prescribed drugs in CF is azithromycin, a 15-membered ring azalide macrolide antibiotic that inhibits bacterial protein synthesis by binding to the 50S ribosomal subunit. Placebo controlled randomized studies in CF have shown that azithromycin treatment is associated with an improvement in lung function, reduced pulmonary exacerbations (PEx), and improvement in nutritional status [8–11]. However, these benefits are more pronounced in CF patients chronically infected with *P. aeruginosa*– a pathogen with high-level intrinsic azithromycin resistance [12]. Accordingly, alternate explanations for this beneficial effect have focused on azithromycin’s immunomodulatory or anti-viral effects. Azithromycin has demonstrated suppression of both neutrophil oxidative metabolism and serum inflammatory markers [13, 14]. In addition, Meyer et al. [15] showed that azithromycin down-regulates inflammatory cytokine production in CF alveolar macrophages and Cigana et al. [16] found that it reduced the expression of the pro-inflammatory chemokines in CF airway epithelial cells. More recently, alternate hypotheses suggesting intrinsic anti-viral effects of azithromycin against common respiratory viruses implicated in exacerbations have been proposed [17–19]– rationalizing the reduced frequency of exacerbations observed in randomized placebo-controlled trials amongst individuals with CF – regardless of underlying chronic airways pathogens [8, 20].

Azithromycin’s antibacterial properties as a potential mechanism for its benefit have again come under scientific study – but rather than its effects on classical CF pathogens such as *P. aeruginosa*, new lines of enquiry are focusing on non-classical components within the airway’s microbiome. Indeed, recent studies have postulated that macrolides may exert at least a portion of their beneficial effects in non-CF bronchiectasis due to anti-microbiome effects [21, 22]. We postulated that a portion of the clinical benefit derived from CF individuals taking azithromycin may relate to its effects on the microbiota of CF sputum - and that microbiota composition may influence therapeutic response. In this study, we sought to determine if azithromycin associates with changes in the microbial community of sputum of a cohort of adults with CF and if there is an association between microbiome structure and subsequent patient outcomes – that might serve as novel biomarker enabling personalization of treatment for future exploration.

## Results

### Patients characteristics as clinical outcome

Thirty-eight patients from our single centre meeting inclusion and exclusion criteria were included in the study (14 males; 24 females), providing 58 pre-treatment azithromycin samples (median − 154 days (interquartile range (IQR) -368- 0) from start day on azithromycin) and 38 post-azithromycin samples (median + 372 days (IQR + 288 − + 420)). Twenty-nine patients with available sputum samples were naïve to azithromycin treatment and nine patients had received remote azithromycin, with a median duration of 5 years (IQR 4–6 years) off drug. In our cohort, we identified 20 patients (52.6%) with all three time points; Pre, Day 0 and Post samples, 17 patients (44.7%) with just Pre and Post sample; and one patient (2.6%) with only Day 0 and Post samples. Patients included in the study had a median age of 25.4 years (IQR 21.2–31.8) at the day of start of azithromycin treatment. Patients in the cohort were started on azithromycin because of concern for clinical deterioration (21/38; 55%), advancing to standard care (29/38; 76%), or both (10/38; 26%). Median annual rate of lung function decline in the entire cohort did not differ following azithromycin initiation: − 1.69%/year (IQR -4.12- + 1.76) pre-azithromycin and − 2.82%/year (IQR -5.5- + 2.21) post-azithromycin (*p* = 0.822).

Of the cohort, 45% [17] were classified as responders. Comparison of patient’s demographic and clinical characteristics at baseline (i.e. Day 0 of azithromycin treatment when available or the pre-treatment sample) between Responders vs non-Responders were similar with respect to age, gender, lung function (both percent predicted forced expiratory volume in 1 s (FEV_1_%) and percent predicted forced vital capacity (FVC)), CF-related co-morbidities and cultured pathogens (*p* > 0.05), and concurrent therapies - with the exception of the use of inhaled corticosteroids (Table 1). Categorization as Responders did not differ based on treatment cohort period from which the samples were collected (A: 54.5%; B: 50%; C: 35.2%, *p* = 0.267). Twenty-eight (74%) of the cohort were chronically infected with *P. aeruginosa* as per the Leeds definition at the time of azithromycin initiation. Rate of FEV_1_ decline did not differ before or after azithromycin initiation in those with chronic *P. aeruginosa* infection relative to those without [Before; − 2.01 (IQR: − 4.35 – 1.59) vs − 0.62 (IQR: − 4.97 – 2.6), *p* = 0.550 or After; − 2.82 (IQR: − 4.89 – 3.36) vs − 2.25 (IQR: − 11.6 – 1.43), *p* = 0.317]. Treatment rationale for azithromycin initiation did not differ in those chronically infected with *P. aeruginosa* versus those not [standard of care; 23/28 (82%) vs 6/10 (60%), relative risk (RR) 1.37 (95% CI 0.8–2.34), *p* = 0.21); concern for clinical decline; 13/28 (46%) vs 8/10 (80%), RR 0.58 (95% CI 0.35–0.96), *p* = 0.14].

**Table 1:** Patient’s demographics and clinical characteristics at baseline^a^ as a function of azithromycin treatment response

	Relative		
	Responder (*n* = 17)	Non-Responder (*n* = 21)	***P***-value
***Demographics***^b^			
Sex (Male:Female)	7:10	7:14	0.739
Age (years)	26.06 (21.9–29.9)	25.2 (20.7–30.4)	0.964
∆F508 / ∆F508	9 (52.9)	10 (47.6)	1
FEV_1_% predicted	44 (32–62)	56 (40–78)	0.270
FVC % predicted	71 (64–87)	87 (67–99)	0.171
Body mass index (kg/m^2^)	20.1 (18.8–21.4)	20.6 (18.9–22.1)	0.402
**CF** ***related diseases***^b^			
Pancreatic sufficiency	2 (11.7)	3 (14.2)	1
CF-related diabetes	4 (23.5)	1 (4.7)	0.152
CF-liver disease	4 (23.5)	2 (9.5)	0.378
Osteopenia/Osteoporosis	5 (29.4)	10 (47.6)	0.326
***Cultured pathogen***^b^			
*Pseudomonas aeruginosa*	13 (76.4)	15 (71.4)	1
*Staphylococcus aureus*	6 (35.2)	7 (33.3)	1
*Haemophilus influenzae*	1 (5.8)	1 (4.7)	1
*Stenotrophomonas maltophilia*	2 (11.7)	1 (4.7)	0.576
*Escherichia coli*	1 (5.8)	1 (4.7)	1
***Therapies***^b^			
Inhaled DNase	12 (70.5)	12 (57.1)	0.506
Inhaled colistin	0 (0)	1 (4.7)	1
Inhaled tobramycin	6 (35.2)	7 (33.3)	1
Inhaled hypertonic saline	5 (29.4)	9 (42.8)	0.506
Proton pump inhibitor	4 (23.5)	9 (42.8)	0.307
Inhaled corticosteroids	15 (88.2)	9 (42.8)	0.006
Long-acting bronchodilator	16 (94.1)	17 (80.9)	0.355
Short-acting bronchodilator	12 (70.5)	15 (71.4)	1
Pancreatic enzymes	15 (88.2)	18 (85.7)	1
Ranitidine	4 (23.5)	1 (4.7)	0.152
CFTR-modulator	0 (0)	0 (0)	–

Fisher exact probability test at a two-tailed or Wilcoxon rank-sum (Mann-Whitney) tests were performed

*CFTR* Cystic fibrosis transmembrane conductance regulator

^a^Variables were taken from the closest or same day from the Day 0 of azithromycin treatment and patients were categorized as Responder or Non-Responder based on the primary outcome definition

^b^Data are presented as n (%) or median (inter-quartile range)

### Overall CF microbiome

**Fig. 1:**
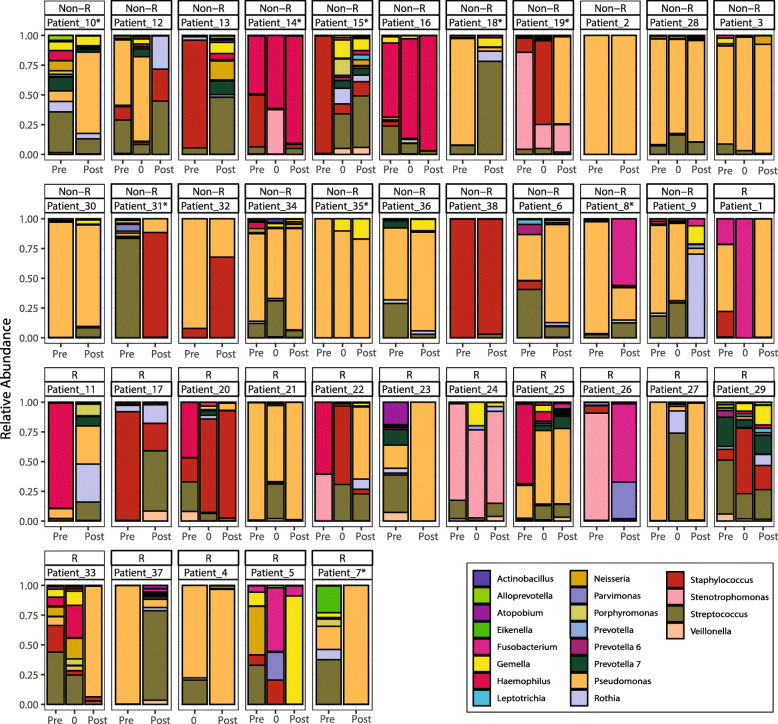
Taxonomic abundance comparison between responders and non-responders to the azithromycin treatment. Relative abundance at the genus level for samples collected at Pre (≤24 months pre-initiation treatment), Day 0 (start day on azithromycin) and Post (≤24 months post its initiation treatment) azithromycin initiation. The top 20 ASVs accounting for > 0.1% of total relative abundance for the whole data set are coloured and presented in the figure. R: responder and Non-responders (NR) to the azithromycin treatment, ASV: amplicon sequence variants. (*) Asterisk in the Patient ID represent patient who were not naïve to azithromycin treatment

A total of 4,705,369 reads (average, 27,659.1 reads/sample; IQR, 19,278 – 72,263.7) with 344 total ASVs (Amplicon Sequence Variant) were recognized. To determine if the diversity observed in our cohort represented the overall diversity, species accumulation curve analysis was performed. We found the number of ASVs increased between 5 and 30 samples and began to plateau by the end of our sampling (See Supplementary Figure 1, Additional file 1). Of the total ASVs found, 20 ASVs (5.81%) accounted for 98.9% of the total number of the reads (Fig. 1). All these ASVs correspond to taxa commonly found in the CF airways communities: *Pseudomonas* (51.2%), followed by *Streptococcus* (12.2%), *Haemophilus* (10.2%), *Staphylococcus* (8.9%), *Stenotrophomonas* (3.7%), *Fusobacterium* (3.0%), *Gemella* (2.4%), *Rothia* (1.8%), *Neisseria* (1.6%), *Prevotella* 7 (1.4%), *Parvimonas* (0.5%), *Veillonella* (0.5%), *Porphyromonas* (0.3%), *Prevotella* 6 (0.3%), *Leptotrichia* (0.2%), *Prevotella* (0.2%), *Alloprevotella* (0.2%), *Atopobium* (0.2%), *Actinobacillus* (0.1%) and *Eikenella* (0.1%).

### Baseline microbiome and primary clinical outcome

**Fig. 2:**
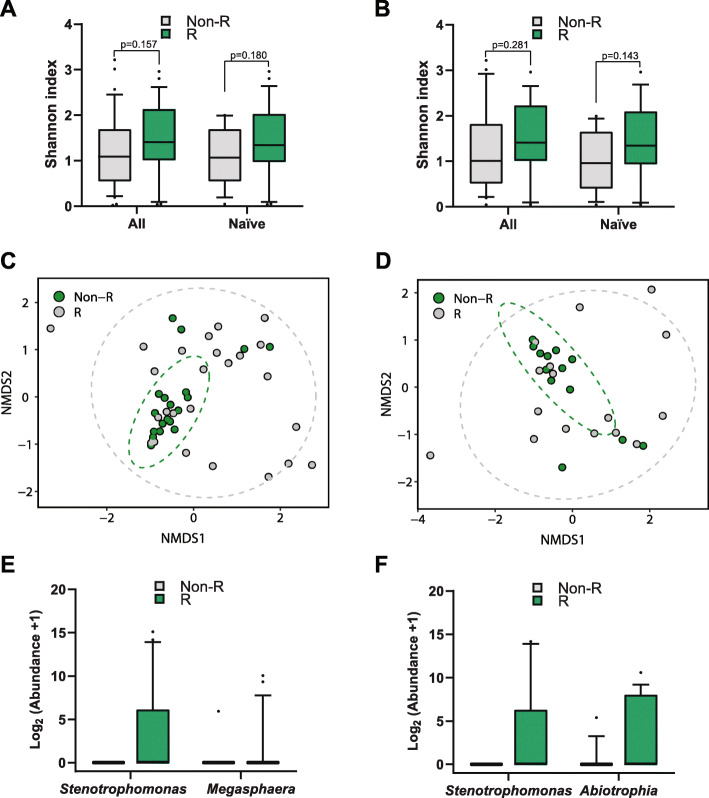
CF microbial community comparison between Responders and Non-Responder to azithromycin treatment. Alpha diversity (within patients) of Responders and Non-Responders based on Shannon diversity index (SDI) metrics for the whole cohort (*n* = 38 patients) and the naïve cohort (*n* = 29) when only the Pre (i.e. Pre and Day 0) (**a**) or baseline samples (**b**) were analyzed. Wilcoxon rank sum test was performed. NMDS plot showing beta diversity of R and Non-R based on Bray-Curtis dissimilarities when only the Pre (i.e. Pre and Day 0) (**c**) or baseline samples were analysed (**d**). ASVs that were identified by DESeq2 to be significantly different (*p* adjusted < 0.05) between Responders and Non-Responders, when Pre (i.e. Pre and Day 0) (**e**) or baseline samples were analysed (**f**), relative abundance is presented in the Log_2_ scale. Boxplots show the median with IQR and the ends of the whiskers mark the 10th and the 90th percentiles. R: responder and Non-responders (NR) to the azithromycin treatment, ASV: amplicon sequence variants

To determine possible biomarkers for response to azithromycin treatment, we compared the alpha diversity from Responders and Non-Responder samples from pre-azithromycin treatment. There were no significant differences in alpha diversity (within-sample diversity) as measured by the Shannon Diversity index (SDI) in Responders relative to Non-Responders for the entire cohort, or exclusively the naïve cohort when measured at either all pre-treatment samples or merely Day 0 samples (Fig. 2a and b). To account for the effect of having multiple samples (i.e. Pre and Day 0) per patient in the SDI between Responder and Non-Responder, we performed linear mixed effects models and found no significant differences in SDI (All cohort: *p* = 0.285, Naïve cohort: *p* = 0.165; Analysis of variance (ANOVA)). Non-metric multidimensional scaling (NMDS) plots based on Bray-Curtis dissimilarity was used to visualise potential clustering among sputum samples based on response-status to azithromycin treatment. Using our relative definition of response, sputum samples clustered by status. However, this was only statistically significant when the naïve cohort was assessed using both Pre and Day 0 samples (*R*^2^ = 7.22, *p* = 0.008, (Permutational multivariate analysis of variance) PERMANOVA) (Fig. 2c) or the baseline samples (*R*^2^ = 7.55, *p* = 0.048, PERMANOVA) (Fig. 2d). Because there were differences in the microbial community structure between Responders and Non-Responders before azithromycin treatment in the naïve cohort, we used DESeq2 to detect ASVs that show significantly differential relative abundance. When both Pre and Day 0 samples were analyzed, we found that *Stenotrophomonas* and *Megasphaera* ASVs were enriched in the responder samples (Fig. 2e) or *Stenotrophomonas* and *Abiotrophia* ASVs when only the baseline samples were analyzed (Fig. 2f) but not with *Pseudomonas* as was expected. As inhaled corticosteroids use differed in our cohorts – we assessed if communities clustered by use at baseline – where no difference was observed when the entire cohort (*R*^2^ = 1.72, *p* = 0.801, PERMANOVA) or the naïve cohort (*R*^2^ = 2.05, *p* = 0.858, PERMANOVA) was analyzed.

### Impact of azithromycin on the CF microbiome

**Fig. 3:**
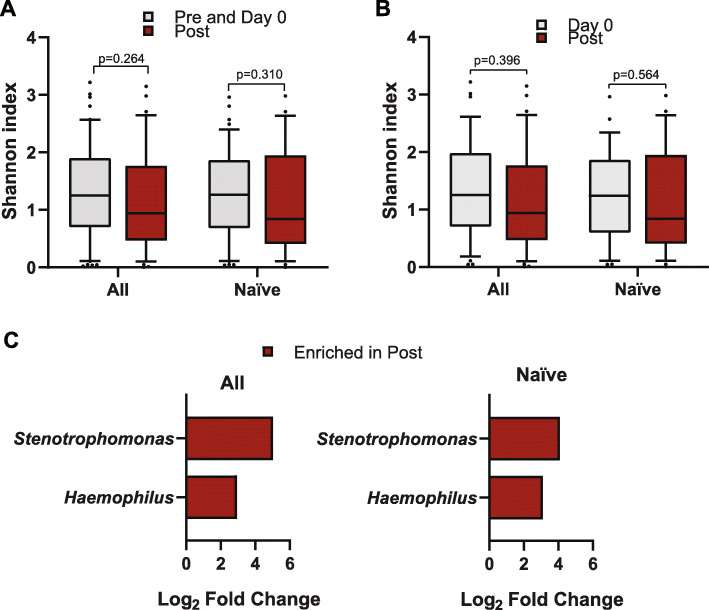
Impact of azithromycin on the CF microbiome. **a** Alpha diversity (within patients) of Pre (i.e. Pre and Day 0) and Post azithromycin samples based on Shannon diversity index (SDI) metrics for the whole cohort (*n* = 38 patients) and the naïve cohort (*n* = 29). Wilcoxon rank sum test was performed. **b** Alpha diversity of baseline and Post azithromycin samples based on SDI metrics for the whole cohort and the naïve cohort. Wilcoxon signed-rank test was performed. Boxplots show the median with IQR and the ends of the whiskers mark the 10th and the 90th percentiles. **c** ASVs that were identified to be significantly different (*p* adjusted < 0.05) in relative abundance between Pre (i.e. Pre and Day 0) and Post groups, as detected by DESeq2

We sought to understand if azithromycin induced large-scale changes in community structure by comparing communities from sputum before and after its use. Based on the Bray-Curtis dissimilarities of sputum samples collected Pre and Post-azithromycin treatment, no differences were observed with either the entire cohort (*R*^2^ = 0.98, *p* = 0.463, PERMANOVA) or when the naïve cohort was tested (*R*^2^ = 0.99, *p* = 0.667, PERMANOVA). Similar results were observed when data was stratified for patient ID (data not shown). Furthermore, no difference in SDI was noted between samples collected before and after azithromycin treatment. This was also true when either both Pre and Day 0 (1.25 vs 0.94, *p* = 0.264) (Fig. 3a) or only Day 0 (1.25 vs 0.94, *p* = 0.396) (Fig. 3b) were compared against the post samples. Similar results were observed when only patients naïve to azithromycin were analyzed (Fig. 3a and b). To account for multiple sampling for the Pre samples (i.e. Pre and Day 0) for some patients, we conducted linear mixed effects models and found similar results (*p* = 0.302, ANOVA).

DESeq2 was used to identify ASVs that differed after azithromycin was initiated. We found *Haemophilus* and *Stenotrophomonas* were enriched in those samples collected after azithromycin relative to before (i.e. Pre and Day 0) when the whole cohort or the naïve cohort was evaluated (*p* adjusted < 0.05) (Fig. 3c). Whereas clustering by azithromycin use was not observed, clustering by traditionally identified factors were. Sputum samples from the same patient were much more similar to each other than to communities of other patients (*R*^2^ = 64.9, *p* = 0.001, PERMANOVA) (See Supplementary Figure 2, Additional file 1). Additionally, communities clustered based on the stage of lung disease (i.e. mild (≥80%), moderate (40–80%), and advanced lung disease: (≤40%)) at the sample collection when it was analyzed the whole cohort (*R*^2^ = 3.45, *p* = 0.045, PERMANOVA) or only the azithromycin naïve cohort (*R*^2^ = 4.99, *p* = 0.03, PERMANOVA).

### CF microbiome following azithromycin initiation treatment

**Fig. 4:**
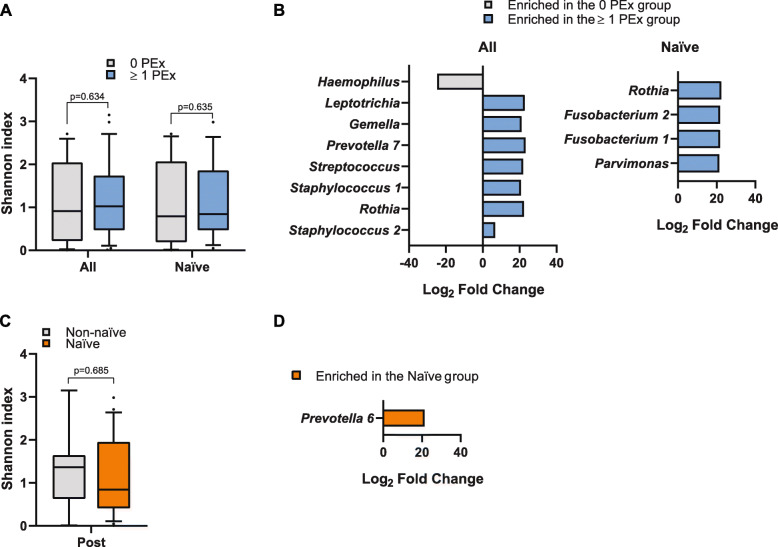
CF microbiome in the Post-azithromycin sputum samples. **a** Alpha diversity (within patients) of samples from patients who experienced one or more than 1 PEx event after azithromycin initiation based on Shannon diversity index (SDI) metrics for the entire cohort (*n* = 38 patients) and those completely naïve (*n* = 29). **b** ASVs that were identified to be significantly different (*p* adjusted < 0.05) in relative abundance between patients with non or more than 1 PEx event during the post period, as detected by DESeq2. **c** Alpha diversity of samples from patients who were naïve to azithromycin or who had not been exposed to azithromycin within at least 3 years. **d** ASV that was identified to be significantly different (*p* adjusted < 0.05) in relative abundance between naive and Non-naïve patients, as detected by DESeq2. For **a** and **c**, Wilcoxon rank sum test was performed, and boxplots show the median with IQR and the ends of the whiskers mark the 10th and the 90th percentiles. ASV: amplicon sequence variants

Review of medical records showed that 27 (71.05%) patients experienced ≥1 clinician defined PEx events in 2 years following azithromycin initiation (1 PEx; 16 (59.3%), 2 PEx; 9 (33.3%) and 3 PEx; 2 (7.4%) patients). We sought to determine if community structure following azithromycin treatment associated with PEx occurrence risk. There was no significant difference for the alpha diversity when it was analyzed in the entire cohort or the naïve subset (Fig. 4a). Similarly, we found that microbial community structure had no association with number of PEx events in the Post samples when the entire cohort (*R*^2^ = 1.60, *p* = 0.851, PERMANOVA) or the naïve cohort (*R*^2^ = 2.64, *p* = 0.637, PERMANOVA) was analyzed. However, when we determined the specific ASVs that showed significantly different relative abundance between having PEx events or not during the Post time, we observed that some taxa, normally found in CF airways communities (i.e. *Haemophilus*, *Streptococcus*, *Prevotella*, *Gemella*, *Staphylococcus*, *Leptotrichia* and *Rothia*), were enriched in patients who exacerbated (Fig. 4b, first panel). Paradoxically, *Haemophilus* was the only taxa whose relative abundance associated with patients who did not experience PEx (Fig. 4b, first panel). In addition to *Fusobacterium* and *Parvimonas*, *Rothia* also appeared to be enriched in the patients that experienced PEx during the post time when the naïve cohort was evaluated (Fig. 4b, second panel). Finally, we evaluated if there were differences in the microbial communities of sputum after azithromycin treatment initiation between patients that were completely naïve to the treatment and those who had not been exposed to azithromycin for three or more years. No significant difference was observed for alpha diversity (Fig. 4c) or beta diversity (*R*^2^ = 2.02, *p* = 0.64, PERMANOVA) in these sub-groups. However, we found that *Prevotella* was enriched in the subset of individuals that were naïve to azithromycin compared to those who were previously exposed three or more years prior (Fig. 4d).

## Discussion

Azithromycin is a critical therapy in CF and has long been included in clinical practice guidelines [23–25]. In those older than 6 years of age with chronic *P. aeruginosa* it is advocated as a recommended therapy to improve lung function and reduce PEx (strength of recommendation B; with a high certainty of net benefit - estimated as moderate) whereas those without chronic *P. aeruginosa* infection azithromycin is recommended to be considered to reduce PEx (strength of recommendation C; with a moderate certainty of net benefit - estimated as mild) [25]. While several investigators now suggest the benefit may be linked to its intrinsic anti-inflammatory or anti-viral properties – a wide variety of studies over the years have sought to identify anti-bacterial links [26, 27]. Indeed, some data suggests that azithromycin is protective against acquisition of new CF pathogens [28]. Owing to the recent recognition of a complex community inhabiting the airways and its association with disease outcomes – others have sought to identify a potential link with azithromycin. Recent work has established that azithromycin induces changes in the sputum microbiome in chronic lung diseases such as asthma [29] and chronic obstructive pulmonary disease (COPD) [30]. Erythromycin, another macrolide antibiotic, was found to induce changes in the microbiota composition in patients with non-CF bronchiectasis [22]. Drawing from this parallel, we hypothesized that azithromycin may induce changes within the CF microbiome - and this may explain some of its associated benefits in CF. However, as azithromycin use in CF (first reported in 1998 [31]) predates the recognition and study of the CF microbiome (first described by Rogers et al. in 2003 [32]), finding treatment naïve patients has proved difficult - limiting the ability of others to perform this important work. Owing to our unique ability to draw from a one-of-a-kind prospectively collected sputum biobank spanning more than 20 years, we were in a unique position to assess for a similar CF-associated effect of azithromycin on sputum microbial community structure of CF adults.

Our primary outcome of interest was to assess if community structure associated with a CF individual’s response to azithromycin treatment (defined by improvement in rate of lung function decline between the start day of treatment and 24 months after it). Identifying signals within the microbiome to serve as biomarkers enabling treatment personalization has been a goal of our group – and one particularly relevant for an agent such as azithromycin where different treatment responses have been observed in clinical trials based on *P. aeruginosa* chronic infection status. In general, we noted no significant differences between Responders and Non-Responders in terms of baseline demographics and clinical characteristics, except for the use of inhaled corticosteroids. We sought to determine whether any microbiome specific biomarkers before azithromycin treatment could be associated with subsequent response to azithromycin treatment. We did not find baseline community structure biomarker as measured by alpha-diversity correlated with azithromycin response as there were no differences in the Pre samples (i.e. Pre and Day 0) between Responders and Non-Responders. We did, however, observe a response-associated community profile based on analysis on the beta diversity using only the cohort of patients that were completely naïve to azithromycin. In particular, we found that genera such as *Stenotrophomonas*, *Megasphaera* and *Abiotrophia* were relatively enriched in those Responders who demonstrated improvement in rate of lung function decline after starting azithromycin. Increased risk of *S. maltophilia* infection have been associated in both pediatric and older CF patients with more advanced lung disease [33, 34].

Our secondary outcome of interest was to assess the frequency of PEx events that patients experience following the initiation of the azithromycin. Unlike changes in lung function, reduction of PEx events has been almost universally associated with azithromycin use – irrespective of baseline pathogens [20]. We found that neither the alpha nor beta diversity differed in the subset of patients who experienced PEx. We did observe some CF community members to be enriched in patients who had PEX after azithromycin initiation. Studies focusing on cultivated pathogens have likewise shown few microbial differences. For example, Samson et al. [35] followed a pediatric CF cohort for 12-months of azithromycin treatment and while they observed reduced PEx, they did not find changes in the prevalence of cultured CF pathogens [35].

We did not find an obvious effect of azithromycin on the CF microbiome in either the naïve or the whole cohort of CF adults. This was somewhat of a surprise as previous studies have shown that azithromycin altered the airway microbiota in other chronic lung diseases such as asthma, COPD and non-CF Bronchiectasis (nCFB) [22, 29, 30]. One possible explanation could be related to the differences of our cohort including, baseline demographics and clinical characteristics, such as age and lung disease stage, both of which are known to have an effect on airways microbiota [36]. In particular, our cohort of CF adults had more advanced lung disease and a microbial community which was much less diverse (median SDI 1.21 (IQR 0.5–1.8) – and thus less likely to experience significant perturbations [37, 38]. It is possible a younger cohort of individuals with CF with more diverse microbial communities – more in keeping with those of asthma, COPD, and nCFB [39, 40] – may be more likely to demonstrate a change. However, other studies focusing on classical CF methods have also found that azithromycin treatment had no major impact on microbiological outcomes in a CF pediatric cohort with early *P. aeruginosa* infection [11] or in sputum bioburden [9]. Although we found that the overall microbial community structure had no association with azithromycin treatment, we found that some specific taxa such as *Stenotrophomonas* and *Haemophilus* were enriched in the Post samples when both the whole and naïve cohort were evaluated. Other work, has previously shown that *H. influenzae* and *S. aureus* were the only two cultured microorganisms that showed an increase in macrolides resistance in a controlled trial of azithromycin in 6–18 years of age CF patients who were uninfected with *P. aeruginosa* [14]. It is important to note that those results were based on qualitative data (i.e. airway culture), since most of the patients included were unable to produce sputum. Lastly, it has been previously shown that CF isolates of *S. maltophilia* are resistant to macrolides – and their recovery following its initiation is thus not unexpected [41, 42].

Several additional limitations of our study warrant consideration. Studies evaluating how CF therapies affect the microbiome can best be done in patients who are naïve to the treatment – a difficult prospect – when studying existing treatments in a population that is already appropriately managed with standard of care treatments like azithromycin. A significant limitation of our study was the modest number of patients in our biobank that met our inclusion and exclusion criteria. This limitation is true to most microbiome studies – where our cohort size is typical. While 76% of our 38 individuals were completely naïve to azithromycin, 9 had prior exposure - a median 5 years earlier. Given the very transient nature of antibacterial effects on the CF microbiome [37, 38, 43, 44] we were comfortable to include those in our study – although we evaluated them both together and separately to account for this. Additionally, although our study had a modest number of patients, when we performed a species accumulation curves analysis to analyze the adequacy of sample size in relation with the bacterial diversity observed, it was found that sequencing depth was appropriate. We suggest that for all future investigational agents that might be perceived to have an effect on the CF microbiome – studies should prospectively collect and store samples during patient follow up enabling the potential for subsequent analysis in a controlled fashion [45]. For example, Segal and collaborators [30] upon exploring azithromycin in COPD were able to assess both the impact on the lung microbiome and metabolome. In order to have a sufficient sample size to analyze amongst a single centre population of adults with CF we utilized samples from three different cohorts of time – introducing cohort heterogeneity given the changes that have been observed over time in CF. We attempted to minimize this by analyzing each cohort together and separately. Furthermore, prior work from our group specifically explored how microbial communities changed over time in different birth cohorts with CF – and observed that the only significant changes in community structure over time related to improving respiratory lung function in successive cohorts [46]. Another limitation of our study is related with the usage of sputum samples as an indicator of the lower airway microbiome. Although studies that use sputum samples have identified the general representation of predominant taxa in the lung microbiota [47], it is important to highlight that some taxa identified in the sputum microbiome could be a result of contamination of oral cavity microbiota.

While the most significant determinant of community clustering in the analysis of serial sputum samples from a cohort is the specific-individual from which a sample is derived [48], increasing data suggests that modest day-to-day changes do exist within an individuals’ expectorated sputum [48, 49]. Accordingly, future efforts to understand the association between specific therapeutics and community structure would benefit from serial sampling – something that was not possible in the retrospective analysis of a clinically collected biobank such as ours despite our efforts to assess pre samples and Day 0 samples separately and together. While we attempted to control for confounders by excluding the most egregious – i.e. parenteral antibiotics [43, 50]– we were not able to control for changes in therapy intrinsic to CF - such as cycled inhaled antibiotics - that can have modest impacts on the CF microbiome in this opportunistically collected biobank of clinical samples [5–7, 51]. Furthermore, increasingly robust data suggests that some of these – notably inhaled tobramycin – may be negatively impacted through azithromycin use (via upregulation of the *mexXY*-efflux pump manifesting in abrogation of tobramycin clinical benefit) – further confounding a treatment-response association that could not be adjusted for in our small study [52, 53]. Inhaled corticosteroid use was found to be more common in our responder population – something that does warrant further exploration given its potential role in respiratory immunomodulation. Despite common usage, the role of inhaled corticosteroids in CF remains uncertain given that there is no clear evidence they reduce inflammation in people with CF or provide clinical benefit [54–56]. Notably, communities did not differ based on their use at baseline. Further studies are required to determine the role of inhaled corticosteroids in individuals with CF and how it may impact response to azithromycin. Indeed, animal models of asthma suggest changes in community structure have been associated with their use [57], whereas no gross changes were observed in humans between high and low dose inhaled corticosteroids [58]. Finally, future studies should not only be focused on how chronic therapies influences the microbiome but also how it influences induction of antimicrobial resistance within different components of the community [59].

## Conclusions

Herein, we found that azithromycin does not appear to induce significant changes in the sputum microbiome of CF adults that were naïve or were not exposed to azithromycin within at least a 3-year period. While samples clustered by patient response status to azithromycin, this effect was small. Our data suggests that the modification of microbial community structure is not a significant mechanism by which azithromycin exerts its clinical benefits in adults with CF.

## Methods

### Patients and sample collection

Patients and samples were retrospectively drawn from a single CF center (the Calgary Adult CF Clinic and its associated Biobank). Patients have prospectively contributed samples to this regional ethics board approved collection from 1998-present (REB15–0854) and provide consent for ongoing research purposes (REB15–2744). This longitudinal collection contains more than 18,000 serial CF sputum samples and has been used for many projects exploring the relationship between the CF microbiome and patient outcomes. Patients were included if they were completely naïve/ or had been off azithromycin for at least 3 years and had at least one sputum before collected and after the initiation of azithromycin. Samples were classified as: before-azithromycin (Pre) (from – 24 months to the day of its initiation), and post-azithromycin (Post) (up to 24 months following initiation treatment). Samples collected at Day 0 (azithromycin start day) were included when available. Sputum samples were excluded from the study if they were collected within 14 days of a PEx and/or the receipt of new systemic antimicrobial agents owing to the potential for confounding effects on the microbiome [43, 50]. Because the study included samples spanning over 15 years – during which many changes in CF care have occurred, samples were categorized in three cohorts based on the year of sample collection: cohort A (2000–2005), cohort B (2006–2010) and cohort C (2011–2015) – and separately analyzed as well. Patient demographics (i.e. sex, age, genotype and nutritional status as measured by body mass index), dynamic variables of disease (i.e. percent predicted forced expiratory volume in 1 s (FEV_1_), percent predicted forced vital capacity (FVC) and CF related diseases), medical therapies and cultured pathogens – at the time of azithromycin initiation - were recorded. FEV_1_ values were normalized using the National Health and Nutrition Examination Survey III references and the FEV_1_% predicted at sample collection was used to categorize patients in the following stages of lung disease: mild (≥80%), moderate (40–80%), and advanced (≤40%).

### Clinical outcome definitions

Treatment initiation rationale was reviewed and rationale for initiation classified as; addition of therapy consistent with standard care, acceleration of treatment owing to clinical deterioration or both. Clinical outcomes in the 2 years before and after azithromycin initiation were collected from a detailed review of each clinical encounter in every patient’s medical record. Dynamic changes in FEV_1_ and PEx occurrence were specifically extracted for each time period. The rate of lung function decline in each period was determined through the construction of subject-specific linear regressions of FEV_1_values collected in those 2 years of study. We included a median of 9 FEV_1_ measurements (IQR 7.2–10.7) per patient, for the 24 months before, and a median of 9 FEV_1_ measurements (IQR 6–10) per patient for the 24 months after in each construct. The primary outcome sought was to differentiate patients based on azithromycin-associated improvements in lung function. Patients were classified as Responders if their net rate of FEV_1_ decline improved (difference between 24 months after treatment and 24 months before treatment is greater than 0) and classified as Non-Responders if no improvement was observed (less than 0). For all the analysis performed in this study, patients were examined in aggregate and a separate sub-group analysis was performed on those patients who were completely naïve to azithromycin. The secondary outcome of interest was the occurrence of PEx. A PEx event was defined clinically as periods of acute worsening with the resultant administration of systemic antimicrobial agents [60]. Clinical charts were retrospectively reviewed, and we verified PEx frequency in the 24 months before and after azithromycin initiation. Patients with chronic *P. aeruginosa* were defined as per the modified Leed’s criteria [61].

### DNA isolation, sequencing and sequence analysis

Total genomic DNA was extracted as previously described [46]. Reagent controls were sequenced and analyzed as quality controls. Barcoded primers were used for paired-end sequencing of the 16S rRNA variable 3 and 4 region using the Illumina MiSeq platform (Illumina, Inc., San Diego, CA, USA) as previously described [4]. After sequencing, adapter and barcode sequences were trimmed using Cutadapt v1.2.1 [62]. Sequences were processed following the Divisive amplicon denoising algorithm (DADA) 2 pipeline (open R package), which sample inference was based on error models constructed for the amplicon dataset [63]. Taxonomic assignments for the non-chimeric amplicon sequence variants (ASVs) table were generated using the Silva SSU r132 reference database [64] and performing the IDTAXA algorithm [65].

### Microbiota analysis

Species accumulation curves (SAC) were determined using the specaccum function of the vegan package in R [66]. Alpha diversity was calculated using the Shannon Diversity index (SDI). Beta diversity was calculated after proportionally normalizing all samples. Beta diversity clustering plots (i.e. non-metric multidimensional scaling (NMDS)) were generated using Bray-Curtis (BC) dissimilarities metrics to visualise potential clustering patterns among samples. Permutational multivariate analysis of variance (PERMANOVA) statistical analysis [67] were performed as previously described [4] to determine factors that may shape the dynamics of the CF microbiome in patients treated with azithromycin. Permutations were stratified to patient ID to account for the random effects of having multiple measures per patient in the pre-treatment category (i.e. Pre and day 0). DESeq2 [68] was used to identify differences in taxa relative abundance among the primary and secondary outcomes and for the impact of azithromycin in the CF microbiome. Taxa with overall relative abundance < 1% were excluded from DESeq2 analysis.

### Statistics

Non-parametric Wilcoxon rank-sum (Mann-Whitney) tests and Fisher exact probability tests were performed for comparisons of patients’ demographics, clinical characteristics, alpha diversity and relative abundance, between Responders and Non-Responders to azithromycin at baseline. Paired statistics (i.e. Wilcoxon signed-rank test) were used for pre-treatment and post-treatment comparisons of changes in alpha diversity and clinical comparisons performed using a two-sided Fisher’s exact test using STATA 16.1 (Stata Corp, Texas, USA). To further examine the effect of multiple samples before treatment (i.e. Pre and day 0) on alpha diversity, linear mixed effects models were generated using the lme4 package in R [69]. We controlled the repeated sampling of the patients by including the patient ID as a random intercept term. False discovery rate was used to control for multiple testing by using Benjamini–Hochberg *P*-value correction in R 3.6.1 (R Core Team, 2019).

## Supplementary Information

**Additional file 1: Supplementary Figure 1.** Species accumulation curves (SAC) determined using the specaccum function from vegan package in R using method = “random” and permutations = 500. SAC plot shows the increase in ASVs detected with the addition of each patient sample. **Supplementary Figure 2.** NMDS plot showing beta diversity of CF patient treated with azithromycin based on Bray-Curtis dissimilarities. Each patient is color coded and was sampled up to three time points: Pre (≤24 months pre-initiation treatment), day 0 (start day on azithromycin) and Post (≤24 months post its initiation treatment). Arrows indicate the timewise sequence of samples (i.e. Pre to day 0 to Post).

## Data Availability

The dataset supporting the conclusions of this article are available in the National Center for Biotechnology Information (NCBI) Sequence Read Archive (SRA) repository, BioProject ID PRJNA666737 (http://www.ncbi.nlm.nih.gov/bioproject/666737). The data will be released to the public when the manuscript is formally accepted for publication. Also, the datasets supporting the conclusions of this article is included within the article and its additional file.

## Abbreviations

ANOVA: Analysis of variance
ASV: Amplicon Sequence Variant
CF: Cystic fibrosis
CFTR: Cystic fibrosis transmembrane conductance regulator
CI: Confidence intervals
COPD: Chronic obstructive pulmonary disease
FEV_1_: Forced expiratory volume in 1 s
FVC: Forced vital capacity
IQR: Inter quartile range
nCFB: Non-CF bronchiectasis
NMDS: Non-metric multi-dimensional scaling
PERMANOVA: Permutational multivariate analysis of variance
PEx: Pulmonary exacerbation
SDI: Shannon Diversity Index
RR: Relative risk
