# Supplementary material for: Azithromycin and the microbiota of cystic fibrosis sputum
Source: BMC Microbiol. 2021 Mar 30;21:96. doi: 10.1186/s12866-021-02159-5 (PMC8008652; doi:10.1186/s12866-021-02159-5)
Supplement: Supplementary file 1 — Additional file 1: Supplementary Figure 1. Species accumulation curves (SAC) determined using the specaccum function from vegan package in R using method = “random” and permutations = 500. SAC plot shows the increase in ASVs detected with the addition of each patient sample. Supplementary Figure 2. NMDS plot showing beta diversity of CF patient treated with azithromycin based on Bray-Curtis dissimilarities. Each patient is color coded and was sampled up to three time points: Pre (≤24 months pre-initiation treatment), day 0 (start day on azithromycin) and Post (≤24 months post its initiation treatment). Arrows indicate the timewise sequence of samples (i.e. Pre to day 0 to Post). [file 12866_2021_2159_MOESM1_ESM.docx]

**SUPPLEMENTARY MATERIAL**

Azithromycin and the microbiota of cystic fibrosis sputum

Nicole Acosta^1^, Christina S. Thornton ^1^, Michael G. Surette^1,3^, Ranjani Somayaji^1,2^, Laura Rossi^3^, Harvey R. Rabin^1,2^ and Michael D. Parkins^1,2*^

Departments of, ^1^Microbiology, Immunology and Infectious Diseases and ^2^ Medicine, University of Calgary, Calgary, Alberta, Canada. ^3^Department of Biochemistry and Biomedical Sciences, McMaster University, Hamilton, Ontario, Canada.


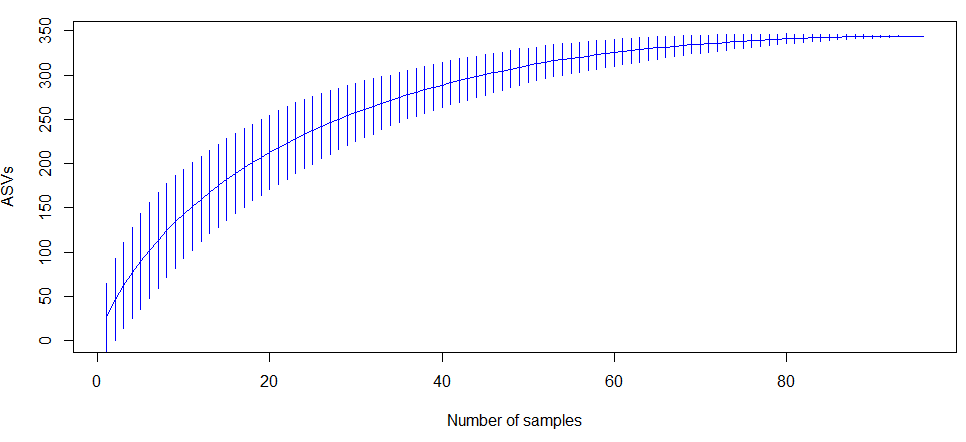


**Supplementary Figure 1.** **Species Accumulation analysis.** Species accumulation curves (SAC) determined using the specaccum function from vegan package in R using method="random" and permutations=500. SAC plot shows the increase in ASVs detected with the addition of each patient sample.

**
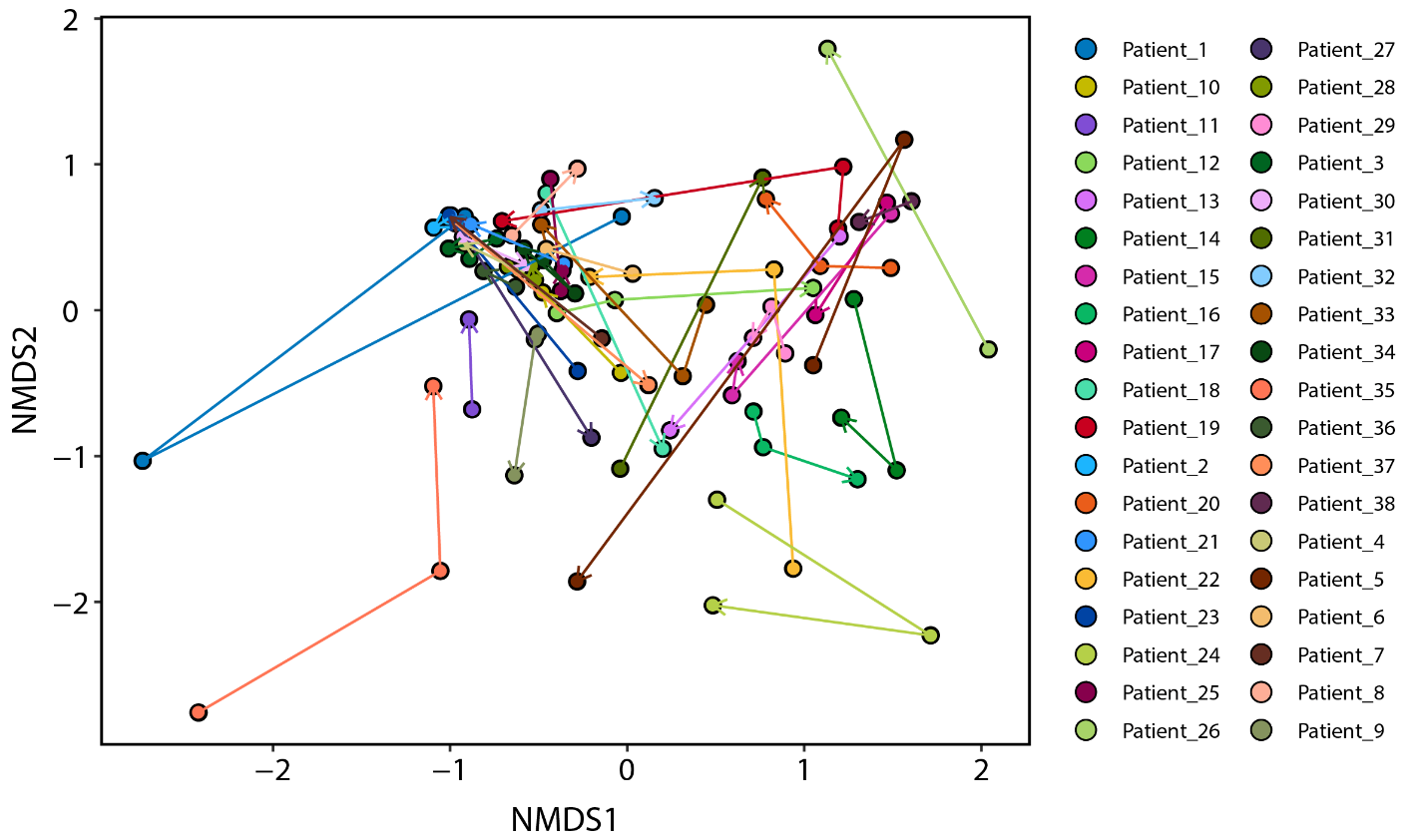
**

**Supplementary Figure 2. CF microbiome is distinguished by patient ID.** NMDS plot showing beta diversity of CF patient treated with azithromycin based on Bray-Curtis dissimilarities. Each patient is color coded and was sampled up to three time points: Pre (≤24 months pre-initiation treatment), day 0 (start day on azithromycin) and Post (≤24 months post its initiation treatment). Arrows indicate the timewise sequence of samples (i.e. Pre to day 0 to Post).
